# Supplementary material for: Biomarkers of Oxidative Stress in Healthy Infants within the First Three Days after Birth
Source: Antioxidants (Basel). 2023 Jun 9;12(6):1249. doi: 10.3390/antiox12061249 (PMC10295668; doi:10.3390/antiox12061249)
Supplement: Supplementary file 1 [file antioxidants-12-01249-s001.zip › antioxidants-2350011-supplementary.pdf]

SUPPLEMENTARY MATERIAL

Table S1. Levels of total antioxidant capacity, creatinine, and malondialdehyde in urine

		0-12 h	12-24 h	24-36 h	36-48 h	48-60 h	60-72 h	>72 h
FRAP	N	53	96	40	101	60	29	14
(mM	Mean	25.08	30.16	37.35	35.89	30.56	30.99	21.76
Fe(II)E)	SD	14.62	17.15	21.30	18.97	18.67	25.47	19.57
	Median	20.8	24.75	38.17	35.28	29.38	25.72	13.57
	IQR	14.48-32.69	15.77-41.22	16.64-52.23	21.27-46.55	13.42-45.15	12.01-39.82	5.96-37.4
Creatinine	N	40	87	34	91	50	26	12
(mg/dl)	Mean	70.49	79.82	95.24	113.05	98.66	84.85	55.04
	SD	52.09	43.57	38.06	55.72	61.23	56.91	53.38
	Median	56.90	72.40	98.00	111.3	95.45	68.50	47.80
	IQR	32.9-90.03	49.5-112.2	61.95-126.53	67.8-148.4	42.28-139.5	39.35-134.45	14.0-62.9
MDA	N	45	91	39	94	55	27	14
(µM)	Mean	1.88	1.82	1.84	1.84	2.04	2.06	1.5
	SD	1.52	1.17	1.09	1.16	1.16	1.28	0.93
	Median	1.12	1.57	2	1.43	2.04	1.51	1.42
	IQR	0.78-2.9	0.94-2.7	0.84-2.74	0.97-2.66	1.08-2.79	0.96-2.78	0.6-2.3

FRAP, Ferric reducing ability of plasma; GSH, MDA, malondialdehyde; SD, standard deviation

Table S2. Correlation between oxidative stress parameters in plasma and urine samples from the study population.

			Urine (<10 h after delivery)		Urine (44 to 52 h after delivery)	
	Blood		TAC	MDA	TAC	MDA
TAC	UV	r	-0.234	-0.001	0.052	0.162
		p-value	0.306	0.997	0.644	0.169
		N	21	16	82	74
	UA	r	-0.547	-0.403	0.107	-0.007
		p-value	0.013[a]	0.136	0.334	0.954
		N	20	15	83	75
	48h	r	-0.185	0.058	-0.037	0.163
		p-value	0.463	0.850	0.730	0.136
		N	18	13	92	85
GSH	UV	r	0.020	-0.058	0.072	0.064
		p-value	0.938	0.844	0.593	0.661
		N	18	14	57	50
	UA	r	0.192	-0.077	-0.012	0.092

		p-value	0.446	0.794	0.934	0.544
		N	18	14	53	46
	48h	r	-0.066	-0.157	0.109	-0.062
		p-value	0.831	0.645	0.392	0.634
		N	13	11	64	61
GSH/GSSG	UV	r	0.180	0.700	-0.006	-0.029
		p-value	0.461	0.004[a]	0.959	0.829
		N	19	15	66	59
	UA	r	0.039	0.373	-0.103	0.041
		p-value	0.877	0.188	0.427	0.769
		N	18	14	62	55
	48h	r	-0.008	0.145	-0.193	-0.032
		p-value	0.975	0.592	0.093	0.790
		N	20	16	77	71
MDA	UV	r	-0.443	0.343	-0,146	-0,168
		p-value	0.066	0.211	0.276	0.238
		N	18	15	58	51
	UA	r	-0.155	0.140	-0.144	0.007
		p-value	0.567	0.648	0.289	0.964
		N	16	13	56	50
	48h	r	-0.585	-0.874	0.069	-0.336
		p-value	0.222	0.126	0.703	0.064
		N	6	4	33	31
Carbonyl groups	UV	r	-0.395	-0.259	0.119	0.015
		p-value	0.105	0.351	0.332	0.909
		N	18	15	68	63
	UA	r	-0.408	-0.180	-0.024	0.219
		p-value	0.104	0.539	0.842	0.080
		N	17	14	73	65
	48h	r	-0.151	-0.402	-0.141	0.110
		p-value	0.657	0.324	0.243	0.388
		N	11	8	70	64

Legend (Table 4). FRAP, Ferric reducing ability of plasma; GSH, total glutathione; GSH/GSSG, relation of reduced/oxidized glutathione; MDA, malondialdehyde; N, number of observations; r, Pearson coefficient; UA, umbilical artery; UV, umbilical vein.

[a] p-value (two-tail) <0.05 is considered statistically significant.
